# Dendronized Silver Nanoparticles as Bacterial Membrane Permeabilizers and Their Interactions With *P. aeruginosa* Lipopolysaccharides, Lysozymes, and Phage-Derived Endolysins

**DOI:** 10.3389/fmicb.2019.02771

**Published:** 2019-12-06

**Authors:** Karol Ciepluch, Kinga Skrzyniarz, Andrea Barrios-Gumiel, Sara Quintana, Javier Sánchez-Nieves, F. Javier de la Mata, Barbara Maciejewska, Zuzanna Drulis-Kawa, Michał Arabski

**Affiliations:** ^1^Department of Biochemistry and Genetics, Jan Kochanowski University, Kielce, Poland; ^2^Department of Organic and Inorganic Chemistry, Chemistry Research Institute “Andrés M. del Río” (IQAR), University of Alcalá, Madrid, Spain; ^3^Networking Research Center on Bioengineering, Biomaterials and Nanomedicine (CIBER-BBN), Madrid, Spain; ^4^Institute Ramón y Cajal for Health Research (IRYCIS), Madrid, Spain; ^5^Department of Pathogen Biology and Immunology, Institute of Genetics and Microbiology, University of Wrocław, Wrocław, Poland

**Keywords:** silver nanoparticles, endolysin, PEGylation, lipopolysaccharide, peptidoglycan

## Abstract

Antimicrobial proteins, like lysozymes produced by animals or bacteriophage lysins, enable the degradation of bacterial peptidoglycan (PG) and, consequently, lead to bacterial cell lysis. However, the activity of those enzymes is not satisfactory against gram-negative bacteria because of the presence of an outer membrane (OM) barrier. Lytic enzymes can therefore be combined with membrane-disrupting agents, such as dendritic silver nanoparticles. Nevertheless, a lipopolysaccharide (LPS), especially the smooth type, could be the main hindrance for highly charged nanoparticles to get direct access to the bacterial OM and to help lytic enzymes to reach their target PG. Herein, we have investigated the interactions of PEGylated carbosilane dendritic nanoparticles with *P. aeruginosa* 010 LPS in the presence of lysozymes and KP27 endolysin to find out the main aspects of the OM destabilization process. Our results showed that PEGylated dendronized AgNPs overcame the LPS barrier and enhanced the antibacterial effect of endolysin more efficiently than unPEGylated nanoparticles.

## Introduction

Multidrug-resistant (MDR) pathogens are an established and growing worldwide public health problem. Among others, the ESKAPE group (*Enterococcus, Staphylococcus, Klebsiella, Acinetobacter, Pseudomonas*, and *Enterobacter*) seems to be the most crucial and particularly dangerous. These are MDR gram-positive and gram-negative bacteria with a broad spectrum of virulence factors causing serious damage to host tissues and efficiently evading the immune system response (Pendleton et al., 2013). Since the variety of MDR bacteria has expanded, therapeutic and prevention options have become limited.

Nowadays, there are several possible strategies to overcome MDR mechanisms, including the application of bacterial viruses (phages). Currently, the interest in lytic phages is constantly growing (Maciejewska et al., 2018). The latest studies showed that phage-derived proteins, especially peptidoglycan (PG)-degrading enzymes (lysins), act as efficient antimicrobials and exhibit an economical potential (Latka et al., 2017; Maciejewska et al., 2018). The PG-degrading effect provided by lysins can be seen in seconds as the osmotic lysis of targeted cell, making these enzymes a desired and efficient antibacterial agent (Nelson et al., 2012; Latka et al., 2017).

It is known that phage lysins, as well as. lysozyme, present a high effectiveness against gram-positive pathogens but have limited activity on gram-negative bacteria due to impeded penetration through the outer membrane (OM) layer. There are several options to improve the antibacterial activity of lysins toward gram-negative bacteria, e.g., using OM destabilizing agents in a mixture or genetically modifying enzymes such as Artilysins (Briers et al., 2014; Peng et al., 2017). Cationic nanoparticles, e.g., dendrimers, are one type of compound that is able to destabilize OM and, in combination with phage endolysin, have improved the antibacterial activity of the mixture (Ciepluch et al., 2019). Additionally, cationic dendrimers are antimicrobial agents itself (Fuentes-Paniagua et al., 2016). The synthesis and further modification of polycationic dendrimers allow for an effective internalization with gram-negative cells (Vankoten et al., 2016). Another class of systems with antibacterial properties are silver nanoparticles (Kim et al., 2007). Silver nanoparticles demonstrated an efficient antibacterial effect; however, the mode of action is still unclear. It is known that AgNPs may kill drug-resistant bacteria and prevent biofilm formation. AgNPs may interact with gram-negative OM, produce reactive oxygen species ROS in bacteria, and the released Ag^+^ ions interact with biomolecules affecting different metabolic pathways, leading to cell death (Qing et al., 2018). AgNPs administered to an animal body can cross the blood–brain barrier as well as accumulate in the liver, spleen, kidney, and brain (Liao et al., 2019).

Both dendrimers and silver nanoparticles (AgNPs) can also be cytotoxic to eukaryotic cells, and they are therefore usually modified with polyethylenoglycol (PEG) to reduce an unfavorable toxic effect and to improve bioavailability and stability (Turecek et al., 2016). Recently, it was shown that dendronization of AgNPs with cationic carbosilane dendrons increases the antibacterial properties of nanoparticles (Peña-González et al., 2017). The activity/toxicity balance was further improved by PEGylation of these dendronized AgNPs. The AgNPs did not induce the resistance in bacteria, and the PEGylated one also prevent biofilm formation at concentrations far below the minimum bactericidal concentration (MBC).

The antibacterial mode of action of these nanoparticles is still unknown. Moreover, the effect of PEGylation of dendronized AgNPs on their interactions with the bacterial OM and lipopolysaccharides (LPSs) have not been tested yet. In our study, we have therefore focused on the PEGylation effect on the interaction between dendronized AgNPs and LPS as well as on the antimicrobial activity of PG-degrading enzymes (lysozyme and KP27 endolysin). An LPS, as a virulence factor, is also a hindrance for many of cationic nanoparticles to reach and destabilize the bacterial OM. Knowledge about the behavior of tested nanoparticles can lead to an assessment of whether PEGylation can improve the antibacterial effect of dendritic AgNPs and enhance antibacterial efficacy of endolysin against gram-negative bacteria. We tested two AgNPs, one dendronized with cationic carbosilane dendrons of the first generation (Dend-AgNP) and the modified version containing PEG ligands on the surface (PEG-Dend-AgNP).

## Materials and Methods

### Dendronized AgNPs Preparation

**FIGURE 1:**
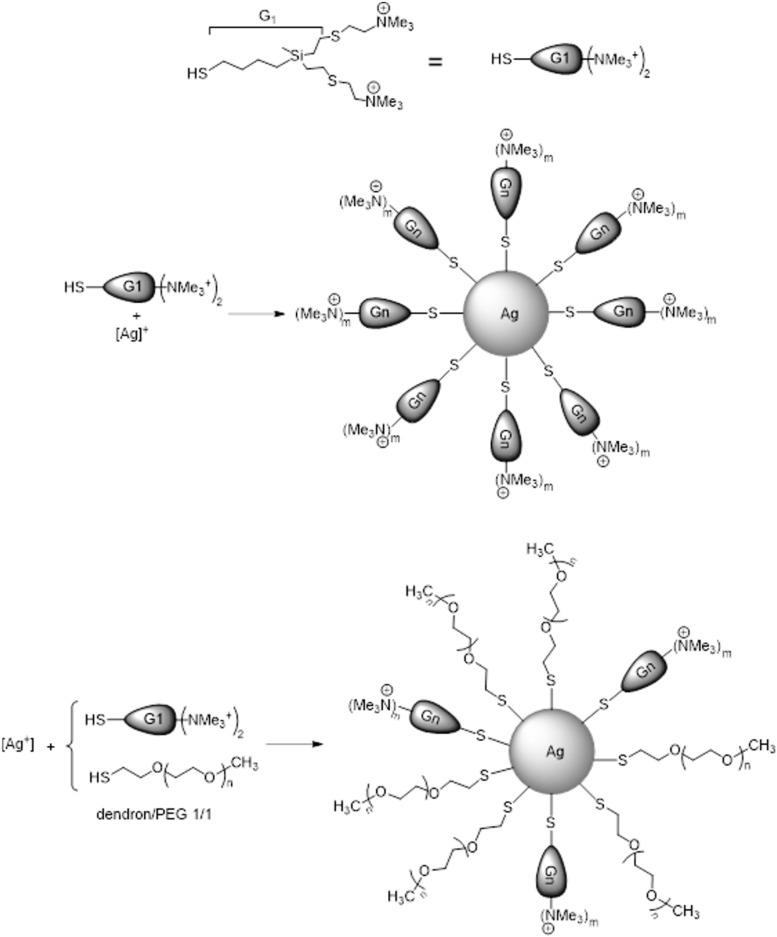
Drawing of first generation cationic carbosilane dendron (top) and reaction schemes of AgNP synthesis (middle, Dend-AgNP; bottom, PEG-Dend-AgNP).

The dendronized AgNPs, Dend-AgNPs (Peña-González et al., 2017), and PEG-Dend-AgNPs (Barrios-Gumiel et al., 2019; Figure 1) used here were prepared following the methodology previously described in Supplementary Material.

### Recombinant Endolysin Preparation

The recombinant phage-borne endolysin was prepared according to the method described previously by Maciejewska et al. (2017). Briefly, the coding sequence of *Klebsiella* phage KP27 endopeptidase was amplified using Pfu polymerase (Thermo Fisher Scientific, Waltham, MA, United States) and cloned into the commercially available pEXP-5-CT/TOPO^®^ TA expression vector (Invitrogen, Thermo Fisher Scientific, Waltham, MA, United States) according to the manufacturer recommendations. The expression was conducted for 18 h at 20°C using *E. coli* BL21 (DE3) pLysS (Agilent Technologies, Santa Clara, CA, United States) and isopropyl-β-D-1-thiogalactopyranoside (IPTG; the final concentration of 0.5 mmol/L) as an inductor of the expression. The recombinant protein was purified from the filtered supernatant by affinity chromatography using NGC medium pressure chromatography systems (Bio-Rad, Hercules, CA, United States) combined with 5-ml nickel columns using Bio-Scale Mini Profinity IMAC Cartridges (Bio-Rad, Hercules, CA, United States) and dialyzed against a PBS buffer. The concentration of purified recombinant enzyme was then determined fluorimetrically (Qubit^®^ Protein Assay Kit, Molecular Probes, Thermo Fisher Scientific, Waltham, MA, United States).

### Dynamic Light Scattering

The dendronized AgNPs particles and the LPS micelles size and size distribution (z-average mean) were measured using the dynamic light scattering (DLS) in a photon correlation spectrometer (Anton Paar Light sizer 500, Austria). The refraction factor was assumed at 1.33 while the detection angles were 15°, 90°, and 175°, and the wavelength was 658 nm. Samples in 10 mmol/L Na-phosphate buffer, pH 7.4, were placed in the plastic cells and measured. The data were analyzed using the Anton Paar software. Three measurements were collected for each experiment and the average was calculated.

### PG Degradation Assay

The enzymatic activity of lysozymes (Sigma-Aldrich, United States) and KP27 endolysin were tested by measuring PG degradation. PG was isolated from *E. coli* ATCC 8739 cells. Briefly, *E. coli* was cultivated under aerobic conditions in a fermenter (BioStat A, Sartorius Stedim Biotech) in nutrient broth (BTL, Poland) under controlled conditions (37°C, pH 7.2–7.4, pO2 70–86%). Cells were harvested at the end of the logarithmic growth phase, centrifuged (5,000 *g*, 30 min), washed with distilled water, and lyophilized. PG was isolated in accordance with the method described by Bera et al. (2005). The rate of degradation was evaluated spectrophotometrically in the presence of dendronized AgNPs. The enzyme (5 μmol/L) was tested alone or preincubated with both types of dendronized AgNPs (PEGylated and unPEGylated one) at the concentration of 30 μg/mL in PBS solution. The 0.25 mg/ml of PG was added to the sample and the kinetics of its degradation was measured at 560 nm using Microplate Reader TECAN Infinite 200 PRO (Tecan Group Ltd., Switzerland) for 80 min. The measurements were done at 37°C. Experiments were repeated twice.

### Protein Secondary Structure Analysis

The potential influence of nanoparticles on enzymes’ secondary structure was measured by Circular Dichroism (CD) at the far-UV region using J-815 CD spectrometer (Jasco, Japan). The experiments were done in 10 mmol/L sodium phosphate buffer (pH 7.4). The concentration of proteins (lysozyme and KP27 endolysin) was 0.5 μmol/L. CD spectra of protein alone and in the presence of nanoparticles were obtained in the range between 260 and 195 nm. Quartz 0.5 cm path length cells (Hellma, Germany) were used for all CD experiments. The CD spectra were taken for protein alone and after adding of nanoparticles at concentration of 14 μg/mL.

### Intrinsic Protein Fluorescence Analysis – Fluorescence Quenching Method

The potential influence of nanoparticles on protein folding and dynamics was studied by the intrinsic protein fluorescence as the fluorescence quenching of tryptophan residues (Stern and Volmer, 1919). Lysozyme and KP27 endolysin were dissolved in PBS at a concentration of 2 μmol/L. An excitation wavelength of 290 nm was used, and the emission was recorded at 350 nm. Next, increasing concentrations of dendronized AgNPs (quencher,Q) were added and fluorescence spectra were recorded. The same experiment was repeated with the addition of LPS at a concentration of 0.01 mg/mL. The experiments were repeated twice.

### Nanoparticle Morphology Analysis by Transmission Electron Microscopy

The potential influence of proteins and LPS on nanoparticle organization (morphology) was verified by transmission electron microscopy (TEM) (Tecnai G2 Spirit, FEI Company). The dendronized AgNPs morphology alone and in the presence of a lysozyme and LPS was visualized. A lysozyme was added to nanoparticles dissolved in 10 mmol/L sodium phosphate buffer (pH 7.4), where the molar ratio of protein:nanoparticles was 10:1. The concentration of nanoparticles and LPS was the same as was described in DLS method. The resulting mixture was placed on a carbon-coated 200-mesh copper grid (Ted Pella, Inc), incubated for 10 min and drained. Samples were negatively stained for 2 min with 2% (w/v) uranyl acetate. Magnification of 60,000× was used to examine dendronized AgNPs morphology.

### Antimicrobial Activity Assay

The antibacterial activity of PG-degrading enzymes combined with dendrimers was tested using *P. aeruginosa* PAO1 wild-type and its knock-out Δ*wbpL* mutant deficient in the biosynthesis of A-band and B-band O-antigens provided by Andrew M. Kropinski from the Laboratory of Foodborne Zoonoses, Guelph, ON, Canada. The antibacterial activity of dendronized AgNPs and KP27 endolysin was measured on an exponentially growing *P. aeruginosa* culture (OD_600_) by spectrometric method at 600 nm. The dendronized AgNPs alone (concentration from 5 μg/mL to 50 μg/mL) and dendronized AgNPs combined with endolysin (5 μmol/L) were added and the effect was expressed as the percentage of *P. aeruginosa* culture growth (optical density OD_600_) compared to the non-treated bacterial culture (control) using Microplate Reader TECAN Infinite 200 PRO (Tecan Group Ltd., Switzerland). Experiments were repeated twice.

## Results

### Interactions of Dendronized AgNPs With LPS

To observe the influence of dendronized AgNPs and PEGylated dendronized AgNPs on tested enzymes in the mimicking OM environment, it was necessary to first characterize the properties (aggregation and size) of these nanoparticles and LPS alone as well as of the nanoparticles/LPS mixture in phosphate buffer solution. The size of tested nanoparticles (measured by Dynamic Light Scattering method) can be presented as a hydrodynamic radius (R*h*), whereas the autocorrelation function informs us about the homogeneity of samples. The experimentally recorded intensity autocorrelation function g^1^(q,t) is directly linked to the theoretically amenable first-order electric field autocorrelation function g^1^(q,t) through the Siegert (1943) relationship:

(1) g(q,t)2=1+Bg(q,t)12

where B(≤1) is an instrumental parameter, and the magnitude of the wave vector, q, is q = (4πn/λ)sin(θ/2), where λ is wavelength of light, n is a reflective index, and θ is the scattering angle.

At 25°C, the correlation functions were found to exhibit two relaxation modes (polydisperse dynamic relaxation):

(2) $$g^{1}\,\left(t\right)=\int_{0}^{\infty }G\,\left(\Gamma \right)\,\exp\left(-\Gamma \,\tau \right)\,d\Gamma$$

where τ is a relaxation time and Γ = q2D. From the formula we were able to calculate the hydrodynamic radii (R*_*h*_*) via the Stokes–Einstein relationship R*_*h*_* = k_*B*_T/6πη_0_D, where k_B_ is the Boltzmann constant, T is temperature, η_0_ is the solvent viscosity, and D is the mutual diffusion coefficient.

**FIGURE 2:**
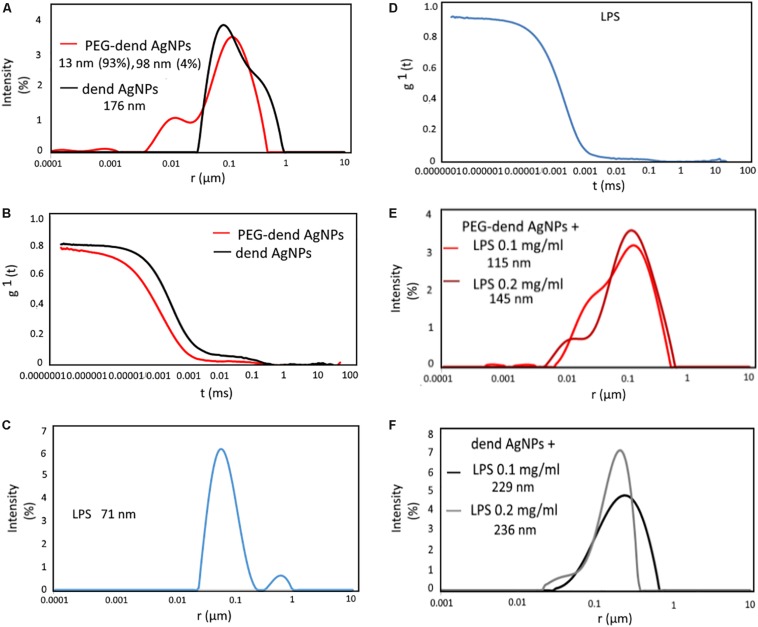
**(A)** Size distribution of dendronized AgNPs and PEG-dendronized AgNPs in PBS buffer (0.01 mol/L) at 25°C. **(B)** Plots of first-order electric autocorrelation function g^1^(t) versus time for dendronized AgNPs and PEG-dendronized AgNPs. For dendronized AgNPs, two relaxation mode (polydisperse dynamic relaxation) was observed. **(C)** Size distribution of LPS in PBS buffer (0.01 mol/L) at 25°C. **(D)** Plots of first-order electric autocorrelation function g^1^(t) versus time for LPS micelles (concentration is 0.1 mg/ml). **(E)** The size distribution of PEG-dendronized AgNPs and **(F)** dendronized AgNPs with LPS (0.1 mg/ml and 0.2 mg/ml) in PBS buffer (0.01 mol/L) at 25°C.

The characteristics of the hydrodynamic radius of dendronized AgNPs (dendronized AgNPs) and PEG-dendronized AgNPs are presented in Figure 2A. In the case of PEG-dendronized AgNPs, two populations of size distribution with a radius of 13 nm and 98 nm could be observed. However, including the volume intensity given by the software, the particles with radius of 13 nm dealt 93% and 98 nm dealt 4% of a total number of particles in solution. The shape correlation function of PEG-dendronized AgNPs presented in Figure 2B was very near to one relaxation mode (homogeneous samples) in contrast to the results obtained for unpegylated dendronized AgNPs. The radius of unpegylated nanoparticles was 176 nm (Figure 2A), and the correlation function was definitely in a two relaxation mode, which suggested heterogeneity of the sample (Figure 2B).

**FIGURE 3:**
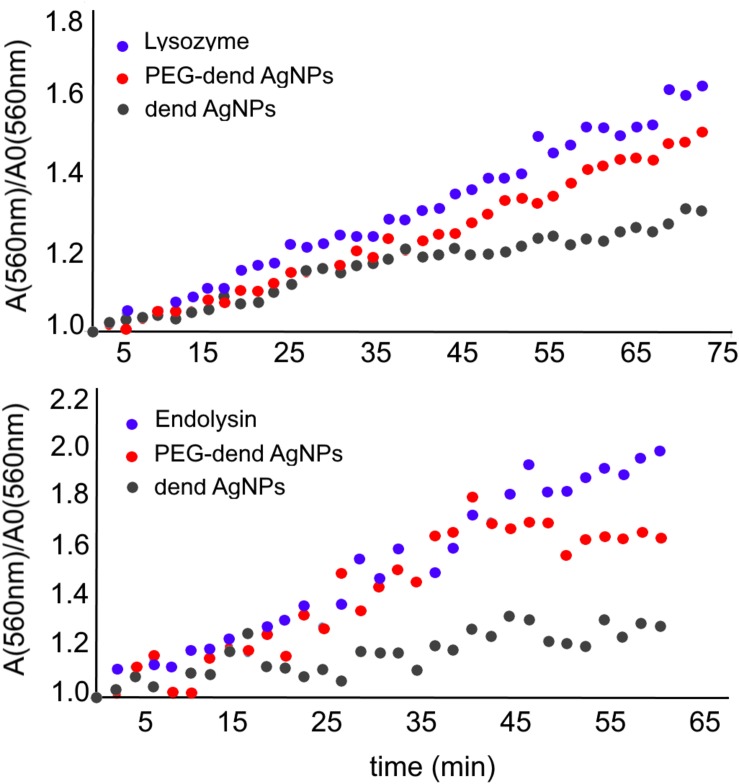
Enzymatic activity of lysozyme and KP27 endolysin in the presence of dendronized AgNPs at 25°C. The activity is described as the ratio of absorbance in time at 560 nm to the absorbance at time *t* = 0. The increase of absorbance means PG degradation by the enzyme in time.

**FIGURE 4:**
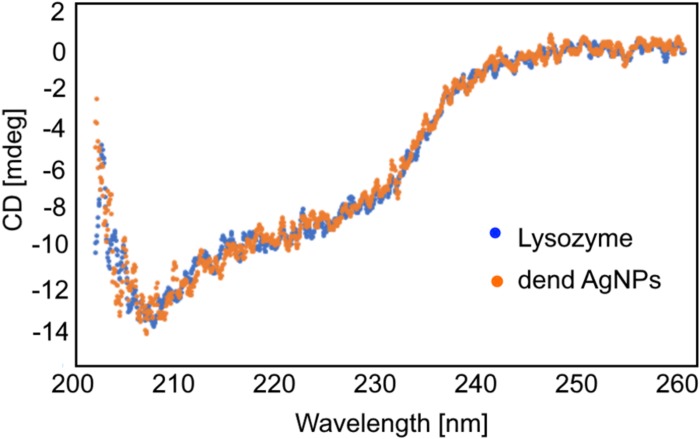
The CD spectra of the lysozyme alone and the lysozyme in the presence of unmodified dendronized AgNPs.

Figure 3 shows the size distribution of the LPS in a buffer solution. Here, two populations were found, one with a radius of 71 nm and the second with a small population around 1,000 nm, suggestive of a small amount of aggregates or dust (Figure 2C). The shape of correlation function means a one relaxation mode (Figure 2D). DLS results for dendronized AgNPs in the presence of LPS micelles are displayed in Figure 4. In Figure 2E, we can see the size distribution of PEG-dendronized AgNPs in the presence of LPS (concentration 0.1 mg/ml or 0.2 mg/ml). The peak with the highest intensity (around 115 nm) indicates the complexation of PEG-dendronized AgNPs with LPS micelles and the lower peak (around 15 nm) is associated with free nanoparticles. At a higher concentration of LPS, the lower peak starts to disappear whereas the higher one increases up to 145 nm. It means that fewer free nanoparticles are present in the solution and more complexes are visible. In Figure 2F, the size distribution of dendronized AgNPs with LPS micelles (0.1 mg/ml or 0.2 mg/ml) is presented. The distribution of peaks is very similar to results obtained for PEGylated nanoparticles. However, the size of the LPS dendronized-AgNPs complex is much bigger (around 229 nm), increasing along the concentration of the LPS (up to 236 nm).

### Interactions of Dendronized AgNPs With Antimicrobial Proteins in the Presence of LPS

The properties of dendritic silver nanoparticles in the presence of LPS are crucial. However, to consider the synergistic effect of NPs together with an antimicrobial protein, the most important issue is to check these interactions also in the presence of an LPS in order to mimic the natural environment of gram-negatives surface macromolecules.

To analyze the influence of dendronized nanoparticles on biological properties of antimicrobial proteins (lysozyme and KP27 endolysin) and their properties in the presence of an LPS as a major component of gram-negative OM, four techniques were applied: PG degradation assay, CD, TEM, and the fluorescence quenching method.

The enzymatic activity of lysozyme and KP27 endolysin in the presence of nanoparticles was measured by PG degradation in a function of time (Figure 3). Since PG is not soluble in water, the absorbance increases over time as an effect of murein aggregates degradation (A/A0), where A0 is the initial absorbance (time *t* = 0 min) and A is the absorbance at a particular time. The addition of PEG-Dend-AgNPs to PG caused a small inhibitory effect on enzyme activity. In the presence of unmodified AgNPs (Dend-AgNPs), the slope of the curve is less steep (higher inhibitory effect) compared to the slopes of a free lysozyme and a lysozyme with PEG-Dend-AgNPs. The enzymatic activity of KP27 endolysin alone and in the presence of AgNPs was measured the same way as for lysozyme. The curve of absorbance dependence in time is steeper compared to that of the lysozyme alone (after 65 min the A/A0 reach 2.0 for endolysin and 1.5 for lysozyme). The addition of PEG-Dend-AgNPs to KP27 endolysin stopped the PG degradation after 40 min of incubation, whereas Dend-AgNPs after 20–25 min.

To check whether the inhibitory effect of dendronized AgNPs (unmodified and modified with PEG) on the activity of enzymes is connected with the structural conformation changes, the measurement of the secondary structure composition of both proteins was carried out. The CD spectra of both enzymes are characteristic for protein with dominant of α-helix structure with two minima at 208 nm and 220 nm. After the addition of excess AgNPs to both protein solutions, no changes in the shape of spectra were observed. An example of the CD spectra of the lysozyme after the addition of dendronized AgNPs is presented in Figure 4. The spectra of KP27 endolysin alone and after the addition of dendronized AgNPs were the same as observed for the lysozyme.

**FIGURE 5:**
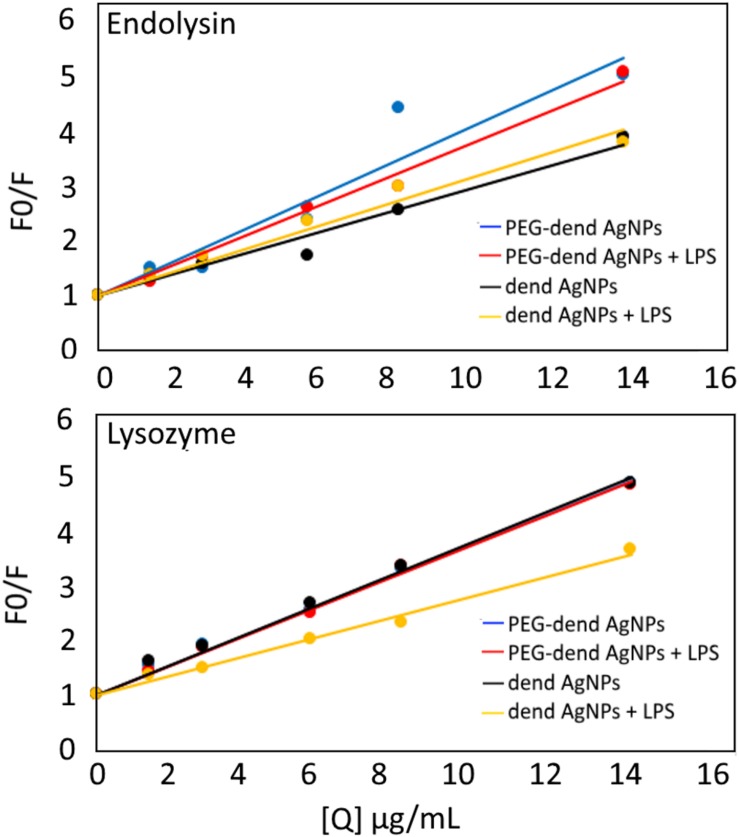
Stern–Volmer plots for protein fluorescence quenching by dendronized AgNPs. λ_*ex*_ = 290 nm and λ_em_ = 350 nm. Q (quencher) means the concentrations of dendronized AgNPs.

The fluorescence quenching of tryptophan residues in proteins was measured to check whether any of the interactions existed between dendronized AgNPs and tested enzymes (Figure 5). A lysozyme contains six tryptophan residues (at positions 28, 62, 63, 108, 111, and 123), while in KP27 endolysin only three tryptophan residues can be found (at positions 79, 86, and 110). The intensity of tryptophan fluorescence in KP27 endolysin decreased with the successive addition of each type of AgNPs (an increase of F0/F, where F0 is fluorescence intensity without nanoparticle and F is intensity after nanoparticles addition). The data was presented as Stern-Volmer plots. The concentration of nanoparticles was from 1 to 14 μg/mL and the concentration of KP27 endolysin was 2 μmol/L. The decrease of fluorescence intensity of KP27 endolysin after PEGylated dendronized AgNPs addition in the presence of an LPS was somewhat smaller compared to the condition without an LPS. This effect was not observed for unmodified dendronized AgNPs in the presence of an LPS. Surprisingly, in this case a decrease of fluorescence intensity was even higher compared to the condition without an LPS. The quenching of tryptophan residues in lysozymes after nanoparticle addition was also measured. No visible differences between the quenching of the fluorescence of lysozymes after the addition of PEG-Dend-AgNPs in the presence of or without an LPS. The difference was observed for unmodified Dend-AgNPs, where a decrease of fluorescence quenching is visible in the presence of LPS. The shape of Stern–Volmer plots for both proteins after nanoparticles addition suggests the typical dynamic (collisional) mechanism, where the contact can be due to diffusive encounters. It is worth mentioning that, for KP27 endolysin, the stronger quenching is visible with the PEGylated nanoparticles combination. However, there is no difference in quenching between PEGylated and unPEGylated nanoparticles incubated with lysozyme.

**FIGURE 6:**
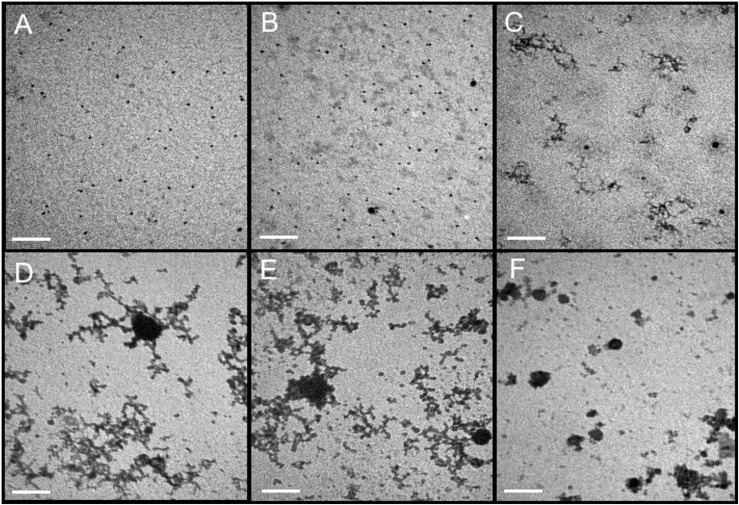
Electron microphotographs of PEGylated dendronized AgNPs **(A)** with lysozyme **(B)** and lysozyme in presence of LPS **(C)**. The morphology of unPEGylated dendronized AgNPs alone **(D)** with lysozyme **(E)** and lysozyme in the presence of LPS **(F)**. The protein/nanoparticles molar ratio was 10:1. Dendrimers and proteins were dissolved in Na-phosphate buffer 10 mmol/L, pH 7.4. The magnification was 60,000×. The bar indicate was 200 nm.

The morphology of dendronized AgNPs in the presence of a lysozyme and an LPS was examined by TEM. Due to the same size and type of interactions of lysozyme and KP27 endolysin, only the lysozyme was tested by TEM. PEGylated AgNPs (Figure 6A) have no tendency to aggregate and they were visible on the TEM grid as a mixture of single particles with a size close to that obtained from DLS (around 15 nm). There are no differences in the morphology of PEG-dendronized-AgNPs in the presence of lysozyme (Figure 6B). The addition of LPS to a protein-nanoparticles mixture changes the morphology of tested nanoparticles (Figure 6C). PEG-dendronized-AgNPs start to aggregate in the presence of LPS and create a “worm-like” structures. UnPEGylated dendronized-AgNPs, due to the high number of cationic groups, have a higher tendency to create aggregates (Figure 6D). In the presence of a lysozyme (Figure 6E), the aggregation effect is even stronger. However, the addition of an LPS caused the formation of smaller aggregates (Figure 6F).

The above measurements clearly showed that PEGylated NPs have less influence on the activity of both antimicrobial proteins. Moreover, both tested nanoparticles did not affect the secondary structure of endolysin and lysozyme. The interactions between dendritic AgNPs and both enzymes are characterized by collisional interactions, without creating bonds between them. It is worth mentioning that these interactions may also depend on the physical properties of nanoparticles, e.g., the tendency to aggregation at a high concentration and in presence of an LPS, as was observed in TEM experiments.

### Dual Antimicrobial Effect of Dendronized AgNPs and KP27 Endolysin on *P. aeruginosa* Cells

In the end we checked whether dendronized AgNPs combined with antimicrobial proteins against *P.aeruginosa* showed the synergistic effect. To verify if the LPS presence affected dendronized AgNPs/protein interactions, two different bacterial strains were used in experiments: a wild type and a mutant lacking an LPS. This experiment let us explain the role of LPS in the synergistic properties of NPs and protein.

**FIGURE 7:**
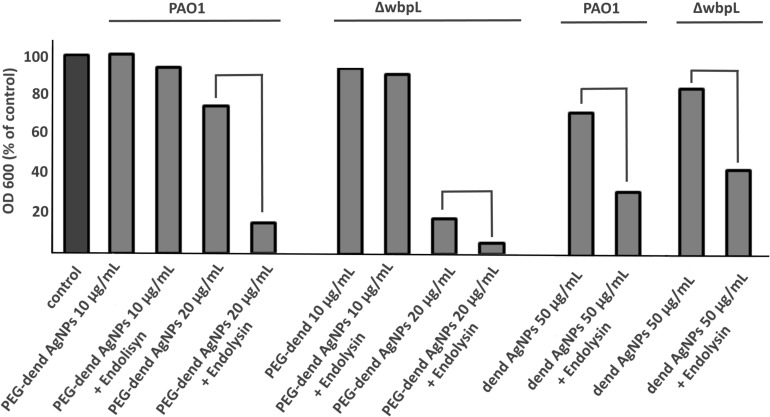
The optical density of PAO1 wild-type and its mutant (ΔwbpL) measured at 600 nm without and in the presence of dendronized AgNPs (at concentrations of 10 μg/mL, 20 μg/mL, and 50 μg/mL) and nanoparticles (the same concentrations) combined with KP27 endolysin (at concentration of 5 μM). The results are expressed as the percentage, where 100% is a non-treated bacterial culture (control). Dend AgNPs means unmodified nanoparticles with PEG. The pair lines indicate the additional effect of endolysin addition. Culture growth for 24 h at 37°C.

Due to the stronger hydrolytic activity of KP27 endolysin in comparison to the lysozyme (Figure 3), we decided to use only the endolysin in this experiment. The dual antimicrobial effect of PEGylated and unmodified dendronized AgNPs combined with KP27 endolysin was evaluated spectrometrically, by measuring the optical density changes of the bacterial culture at 600 nm. Bacterial strains differed in the length and type of LPS O-chain, and the influence of nanoparticles with or without endolysin on wild-type *P. aeruginosa* PAO1 and its LPS knock-out mutant (Δ*wbpL*) was therefore analyzed (Figure 7). For the experiment, different concentrations of nanoparticles (5–50 μg/mL) and a constant concentration of KP27 endolysin (5 μmol/L) were used. No significant decrease in OD was observed for both tested strains treated with PEGylated nanoparticles at a concentration of 10 μg/mL. The addition of endolysin in this condition leads to a decrease of OD by several percent compared to untreated bacteria and bacteria treated with 10 μg/mL of nanoparticles only. A higher reduction of OD (by 25% and 82% for PAO1 and Δ*wbpL*, respectively) was observed in the presence of PEGylated nanoparticles at the concentration of 20 μg/mL. The antibacterial effect after addition of unmodified dendronized AgNPs was also reached at a concentration of 50 μg/mL. The addition of endolysin led to a further decrease of OD of PAO1 and Δ*wbpL* mutant (from 70% to 30% and from 80% to 40%, respectively). Obtained results clearly showed a synergic effect of PEgylated dendronized AgNPs and endolysin against gram-negative bacteria.

## Discussion

There are numerous research works about the antibacterial effect of dendronized nanoparticles, e.g., dendrimers and AgNPs (Gholami et al., 2017; Peña-González et al., 2017). The mixture of metal nanoparticles with dendrimers may be very promising for the development of new antimicrobial systems (Mignani et al., 2017; Peña-González et al., 2017). It is known that antibacterial properties of nanoparticles (both dendrimers and silver NPs) strongly depend on their structure, charge, size, and adsorption ability at the bacterial surface (Felczak et al., 2012; Le Ouay and Stellacci, 2015; Tang and Zheng, 2018). As potential membrane permeabilizers, the dendronized nanoparticles may also enhance the antibacterial effect of antibiotics or antimicrobial proteins, especially against gram-negative bacteria (Winnicka et al., 2013; Serri et al., 2019). Our recent work showed that poly(propylene) imine glycodendrimers improved the antimicrobial properties of phage KP27 endolysin against *P. aeruginosa* cells (Ciepluch et al., 2019). Our studies suggested that cationic charged nanoparticles interacted strongly with bacterial surface glycans like LPS, and those interactions should be taken into account in the design of new antibacterial tools.

Negatively charged LPS is a major element affecting the antibacterial effect of cationic nanoparticles. Therefore, one of the aims of this study was to test the influence of dendronized AgNPs pegylation on their interactions with *P. aeruginosa* LPS. Understanding the nature of these interactions in solution may give very important information in the context of developing new antibacterial tools. DLS technique provides information about the aggregation of nanoparticles alone and the size of an eventual complex between nanoparticles and LPS micelles. The obtained results indicated that unPEGylated Dend-AgNPs have more of a tendency to aggregation compare to PEGylated PEG-Dend-AgNPs. Perhaps this is connected with the hydrophobic/hydrophilic balance of DendAgNPs. The PEGylation decreased the cationic charge of not only the nanoparticles surface but also the number of dendrons, to which the inner structure is highly hydrophobic; these ligands are being substituted by polar PEG moieties, consequently leading to the decrease of aggregation.

The increasing size of dendronized silver NPs (PEGylated and unPEGylated ones) in the presence of LPS, suggested that “LPS: dendronized Ag-NPs corona” may occur during the complexation event. The pegylated NPs do not aggregate, and thus this peak comes from the complex of PEG-dendronized AgNPs and LPS. It is very hard to distinguish separate peaks in a mixture of unPEGylated NPs and LPS micelles; therefore, we may say that both complexes of NPs and LPSs and aggregates of free NPs were formed. We can speculate that the mechanism of unPEGylated NPs binding to LPS seems to be different compared to PEGylated nanoparticles. It is worth mentioning that PEGylated dendronized AgNPs can create a complex with LPS (visible on one broad peak), but the effect of creation the LPS-unPEGylated NPs corona is not favored due to the aggregation tendency of unPEGylated dendronized AgNPs.

The influence of dendronized AgNPs on biological properties of antimicrobial proteins (lysozyme and KP27 endolysin) and their properties in the presence of LPS seems to be crucial as well, especially in the context of the dual antimicrobial effect. First, the effect of dendronized AgNPs on the structure and activity of lysozymes and KP27 endolysin was checked. Circular dichroism data clearly show that tested nanoparticles do not change the secondary structure composition of proteins. However, the enzyme activity assay suggests that dendronized AgNPs inhibit the activity of lysozymes and endolysin. In both cases, unPEGylated nanoparticles have a stronger influence on enzymes activity. Interestingly, both nanoparticles finally inhibit completely the activity of KP27 endolysin but not of lysozymes. This could be due to the differences in shape, the charge of the active site and the isoelectric point of the lysozyme and KP27 endolysin. Although the charge of the tested lysozyme at pH 7.4 is slightly more positive (isoelectric point is more than 10) compared to endolysin, it is unlikely to expect a “stiff” complex between the protein and NPs. In this case, we should expect rather collisional interaction (where the contact between the protein and nanoparticles can be due to diffusive encounters), which is very common for AgNPs (Wang et al., 2017; Ashrafpour and Tohidi Moghadam, 2018). Our fluorescence data also showed the collisional dynamic interaction between dendronized AgNPs and proteins. However, PEGylated NPs affect KP27 endolysin more (not for lysozyme) compared to unPEGylated ones. The presence of LPS decreased the strength of interaction between NPs and proteins, but the effect depended on the type of protein. The changes in the Stern–Volmer plots were small, but in the case of lysozymes and unPEGylated NPs they were much bigger, suggesting that the LPS has to disturb in collisional interaction between NPs and protein. Due to the negative charge, the LPS molecules would probably compete with the protein to interact with the nanoparticles surface. It may indicate that the LPS could capture the nanoparticles from the solution, leaving more protein particles free and not affected by nanoparticles.

It is known that PEGyaltion of nanoparticles may stabilize the structure and prevent the aggregation (Shkilnyy et al., 2009) due to steric repulsion (Chang et al., 2016). Therefore, we should expect no aggregation of PEGylated dendronized AgNPs in the presence of lysozymes and the LPS. However, there were no differences in PEG-dendronized-AgNPs morphology in the presence of lysozymes, in contrast to the LPS/protein mixture. This may suggest that PEGylated nanoparticles did not easily bind the protein (lysozyme) to the dendronized surface, but they can bind to the LPS. This corresponds to data obtained from the DLS and fluorescence quenching method, where the interactions between LPS and PEGylated AgNPs occurred. What is very interesting is that, in the case of unPEGylated AgNPs, bigger aggregates were visible in the presence of the lysozyme. This is not surprising because AgNPs can bind lysozymes on their surface (Wang et al., 2017). The data obtained from fluorescence quenching and enzyme activity definitively prove that the lysozyme dendronized-AgNPs complexation. At the same time, smaller aggregates were visible in the presence of LPS. This is somehow an unexpected behavior. The addition of negatively charged LPS leads to the increase of aggregate, as was visible in the DLS. It seems that unPEGylated AgNPs may bind the LPS, but this complex may change the charge of the dendronized AgNPs surface so that more neutral and, thus, smaller aggregates are visible when evaporating a buffer while the TEM grid is prepared. Unfortunately, it does not explain whether the lysozyme is still bound to the nanoparticles surface or not. The fluorescence quenching results suggested that the LPS reduces the influence of dendronized AgNPs on lysozymes, making the quenching smaller. Despite forming smaller aggregates of lysozyme-dendronized AgNPs in presence of LPS, the lysozyme seems to be still present on NPs surface.

The biophysical studies of AgNPs interactions with proteins and/or LPS, suggest that the PEGylation may completely change nanoparticle properties. The interaction process with LPSs or proteins depends on the cationic charge of the macromolecules. The tendency toward aggregation is strong but can change in the presence of an LPS or in the presence of an antimicrobial protein. The PEG chain connected to the surface may turn properties of nanoparticles or other compounds in a different way (Chang et al., 2016; Shen et al., 2018). This was observed in an antimicrobial assay done on two *P. aeruginosa* strains (with an LPS and without). The higher antimicrobial effect as membrane permeabilizers was obtained for PEGylated dendronized AgNPs alone versus with unmodified nanoparticles. The enhanced antimicrobial effect was also achieved in combination with KP27 endolysin. The synergic effect was seen in the presence of PEGylated and unPEGylated dend-AgNPs at the concentration of 20 μg/mL and 50 μg/mL, respectively. Unexpectedly, a lower cationic charge of PEGylated particles allowed for OM permeabilization at a lower concentration compared to unPEGylated molecules. We hypothesized that cationic unmodified dendronized AgNPs aggregated at the bacterial membrane (or in the solution), blocking the PG-degrading enzyme from gaining access to the target PG. The PEGylation prevented this undesirable effect. The pegylated nanoparticles showed a stronger synergic antibacterial effect in combination with KP27 endolysin against the LPS-deficient *P. aeruginosa* mutants compared to the wild-type bacteria. In contrast, unPEGylated NPs were equally effective on both *P. aeruginosa* strain. The similar effect of unPEGylated NPs on WT and mutant strains could be due to properties of nanoparticles. The aggregation of unPEGylated NPs may also occur not only in the presence of LPS but also at a high concentration of unPEGylated NPs alone. At a high local concentration of NPs, the attractive interactions between NPs are very common, and thus the aggregation is still possible, even in the absence of an LPS.

## Conclusion

In conclusion, both PEGyled and unPEGylated dendronized AgNPs are efficient and permeabilize gram-negative bacteria OM to allow the phage endolysin to reach the target PG. Nevertheless, the PEGylation modification increases the OM permeabilizing effect, overcoming the LPS barrier at a lower concentration. This study may bring a new light to the designing of the new tools composed of PG-degrading enzymes like phage lysins and OM permeabilizers, such as dendronized AgNPs, and toward the future development of a satisfactory antibacterial agent against gram-negative bacteria.

## Data Availability Statement

All datasets generated for this study are included in the article/Supplementary Material.

## Author Contributions

KC, KS, MA, and ZD-K contributed to the conception and design of the study. AB-G, SQ, JS-N, and FM synthesized the nanoparticles. BM and ZD-K obtained the recombinant endolysin. All authors contributed to the manuscript revision, and read and approved the submitted version.

## Conflict of Interest

The authors declare that the research was conducted in the absence of any commercial or financial relationships that could be construed as a potential conflict of interest.
