# Supplementary material for: Dendronized Silver Nanoparticles as Bacterial Membrane Permeabilizers and Their Interactions With P. aeruginosa Lipopolysaccharides, Lysozymes, and Phage-Derived Endolysins
Source: Front Microbiol. 2019 Dec 6;10:2771. doi: 10.3389/fmicb.2019.02771 (PMC6908850; doi:10.3389/fmicb.2019.02771)
Supplement: Supplementary file 1 [file Data_Sheet_1.PDF]

Supplementary Material

1 Supplementary Data

General considerations

All reactions were carried out under inert atmosphere and solvents were purified from appropriate drying agents when necessary. Unless otherwise stated, reagents were obtained from commercial sources and used as received. Dendron $HSG_1(S\text{-}NMe_3Cl)_2$ was synthesized as published.[1]

Synthesis of nanoparticles

AgNP@(SG₁(S-NMe₃Cl)₂) (Dend-AgNP). These AgNP were synthesized as described elsewhere.[2] An aqueous solution of compound $HSG_1(S\text{-}NMe_3Cl)_4$ (**1**) (40 mL, 0.5 mmol, 12.5 mM) as added dropwise to an aqueous solution of $AgNO_3$ (16.3 mL, 0.5 mmol, 30 mM) w. $NaBH_4$ in water (13.5 mL, 2.7 mmol, 200 mM) was next added dropwise, and the mixture stirred for 4 h. Nanoparticles were purified by dialysis (MWCO 10.000) yielding **Dend-AgNP** (108 mg), which were stored in deionized water at 4 ºC.

Data for **Dend-AgNP**: NMR (D_2O): ^1H NMR: δ 0.06 ($SiCH_3$), 0.60 ($SCH_2CH_2CH_2CH_2Si$), 0.90 ($SiCH_2CH_2S$), 1.40 ($SCH_2CH_2CH_2CH_2Si$), 1.78 ($SCH_2CH_2CH_2CH_2Si$), 2.74 ($SiCH_2CH_2S$), 2.97 (SCH_2CH_2N), 3.10 (NCH_3), 3.50 (SCH_2CH_2N). Ag/(l) reactant molar ratio = 1:1. TGA (%): Ag, 46.4; (l), 53.6. Calc. molar ratio Ag/(l) = 3.99:1 in nanoparticle. SPR (UV-Vis): 447.8 nm. Zeta Potential: +53.4. DLS (Z-average d.nm) = 11.70 nm. Mean diameter of silver core (TEM): D = 1.70 nm. Number of silver atoms: N_{Ag} = 143; number of dendrons N_d = 36. Molecular formula: $Ag_{143}(C_{19}H_{45}Cl_2N_2S_3Si)_{36}$. Average Mw = 64733309.85 $gmol^{-1}$.

AgNP@(SG₁(S-NMe₃Cl)₂@PEG (1/1) (PEG-Dend-AgNP). These AgNP were synthesized as described elsewhere.[3] A mix of aqueous solution (24 mL, 12.5 mM) of $HSG_1(S\text{-}NMe_3Cl)_2$ (0.15 mmol,

74.6 mg) and HS-PEG (Mn = 800, 0.15 mmol, 120 mg) was added dropwise to an aqueous solution of $AgNO_3$ (10 mL, 30 mM, 0.3 mmol, 50.9 mg). Afterwards, a solution of $NaBH_4$ in water (7.5 mL, 200 mM, 1.5 mmol, 56.7 mg) was added dropwise and the mixture was stirred for 4 h at room temperature. Nanoparticles thus obtained were purified by dialysis (MWCO 10 kDa) yielding **PEG-Dend-AgNP** (74.7 mg), which were stored in deionized water at 4 ºC.

Data for **PEG-Dend-AgNP**: NMR (D_2O): ^{1}H NMR: δ 0.17 (SiCH_3), 0.73 ($SCH_2CH_2CH_2C$$H_2$Si), 1.03 (SiC$H_2$$CH_2$S), 1.53 ($SCH_2CH_2C$$H_2$$CH_2$Si), 1.91 ($SCH_2C$$H_2$$CH_2CH_2$Si), 2.83 ($SiCH_2C$$H_2$S), 3.11 (SC$H_2$$CH_2$N), 3.22 (NC$H_3$), 3.41 (O*Me*, PEG), 3.66 ($SCH_2C$$H_2$N), 3.73 (OC$H_2CH_2$O, PEG). Reactant molar ratio $HSG_1(S\text{-}NMe_3Cl)_2$/HS-PEG molar ratio= 1/1. Calc. molar ratio (NMR) $HSG_1(S\text{-}NMe_3Cl)_2$/HS-PEG = 1/2.4. TGA (%): Ag, 35.7; L, 64.3. SPR (UV-vis): 442 nm. Zeta Potential (mV): 22.5. DLS (nm): 21.7. Mean diameter of silver core (TEM) = 4.1 nm.
